# Perceived Stress Levels among Ukrainian Migrant and LGBT+ Minorities in Poland during the COVID-19 Pandemic

**DOI:** 10.3390/ijerph182312838

**Published:** 2021-12-06

**Authors:** Tomasz Michalski, Maciej Brosz, Joanna Stepien, Karolina Biernacka, Michal Blaszczyk, Jakub Grabowski

**Affiliations:** 1 Department of Regional Development, Faculty of Social Science, University of Gdansk, 80-309 Gdansk, Poland; tomasz.michalski@ug.edu.pl; 2 Institute of Sociology, Faculty of Social Sciences, University of Gdansk, 80-309 Gdansk, Poland; maciej.brosz@ug.edu.pl; 3 Department of Socio-Economic Geography, Faculty of Social Science, University of Gdansk, 80-309 Gdansk, Poland; joanna.stepien@ug.edu.pl; 4 Adult Psychiatry Scientific Circle, Division of Developmental Psychiatry, Psychotic and Geriatric Disorders, Department of Psychiatry, Faculty of Medicine, Medical University of Gdansk, 80-210 Gdansk, Poland; kbiernacka@gumed.edu.pl (K.B.); michalb079@gumed.edu.pl (M.B.); 5 Division of Developmental Psychiatry, Psychotic and Geriatric Disorders, Department of Psychiatry, Faculty of Medicine, Medical University of Gdansk, 80-210 Gdansk, Poland

**Keywords:** SARS-CoV-2, mental health, depression, stigma, sexual and gender minorities, men who have sex with men, homosexuals, bisexuals, transients, migrant workers

## Abstract

The Coronavirus disease 2019 (COVID-19) pandemic, immigrant status and being a member of the LGBT+ community are all independent factors associated with increased stress levels. Few studies provide more complex analysis on this issue, and there has been no research on the cumulative burden of perceived stress that people belonging to both minorities experience in the current epidemiological situation. The aim of this study was to assess the ability to deal with an external situation during the third wave of the COVID-19 pandemic in Poland in the following groups with different stress levels (total sample *n* = 370): Polish heterosexual men (*n* = 202), heterosexual men from Ukraine (*n* = 131) and homo- and bisexual men (men who have sex with men—MSM) from Ukraine (*n* = 37). A Perceived Stress Scale (PSS-10) was used. The analysis of the survey did not show statistically significant differences between the three study groups in the general level of perceived stress (24.71, 24.77 and 26.49 points, respectively, *p* = 0.551), but it revealed numerous differences in coping with various aspects of everyday functioning between these groups. Negative assessment of one’s own health proved to be the main factor negatively affecting the level of perceived stress, however specific health risks, medical history or the participants’ previous experience have not been taken into account in the study. Our research shows differences in the needs, resources and methods of coping with stress between men who are Polish citizens and migrants from Ukraine, both heterosexual and belonging to the MSM group. Proper identification and addressing of these needs, taking into account different availability of health services, could be the responsibility of NGOs or insurance providers. This should result in the reduction of mental health burdens and the risk of developing serious mental disorders, and consequently in better functioning of persons belonging to minorities and in a reduced burden on the health care system.

## 1. Introduction

### 1.1. Coronavirus Disease 2019 (COVID-19) Pandemic and Mental Health Burden

The COVID-19 pandemic as a threat to global public health has an impact on the mental health and stress levels of different populations. Numerous studies have shown a general deterioration of mental well-being in the general population [1,2]. An increased frequency of symptoms of anxiety, depression and stress disorders has been observed in populations of countries affected by the pandemic, which, according to Stueck’s Pandemic Management Theory [3], follows from the complex mechanism behind facing adversities individually and collectively within a community.

During the COVID-19 pandemic, also in the Polish population, a high level of stress experienced by the studied individuals has been observed [4,5], with a tendency to correlate with factors such as an unstable work situation, a lower level of education, and a younger age. Among residents of Gdańsk (the largest city in the Pomeranian Voivodeship and one of the largest cities in Poland), almost half of the 1500 respondents in non-representative, convenience sampling assessed their mental well-being in June 2021 as worse than before the pandemic [6].

### 1.2. Ukrainian Migrants in Northern Poland

Regardless of the ongoing pandemic, immigrants are known to have a higher level of psychosomatic and mood problems [7]. There is a clear influence of both personality traits and external factors resulting from cultural differences, including the often-different work culture. For migrants, negative consequences include, among others, separation from family and friends (sometimes resulting in family breakdown), living and working in worse conditions in the destination country than in the country of origin or taking up employment inconsistent with qualifications (the so-called brain waste) [8,9].

**Figure 1:**
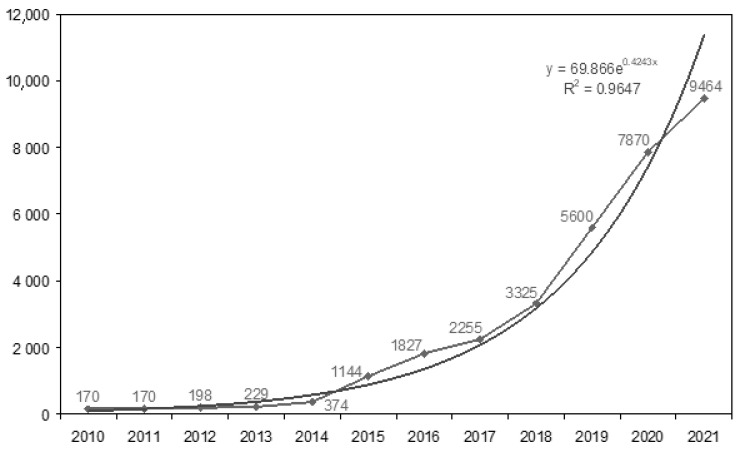
Increase in male Ukrainians with valid documents entitling them to stay in Poland (issued by the Pomeranian voivode). Exponential autoregression (R^2^ = 0.96). Own elaboration of data from Polish Office for Foreigners [15].

Almost all immigrants from Ukraine to Poland are labor migrants. Surveys conducted in the Pomeranian Voivodeship in 2019 show that before the outbreak of the pandemic, immigrants from Ukraine worked mainly in industry, trade, construction and repair services (56.7%). Almost half of the respondents (47.0%) declared that they had been staying in Poland for 1 to 3 years [10]. The large volume of emigration from Ukraine results from a relatively unfavorable economic situation in that country [11]. On the other hand, the fact that Poland, rather than richer countries in the European Union, is most often their country of choice can be explained by cultural proximity and by the fact that only in Poland can citizens of Ukraine work without a visa, provided they have a biometric passport and a declaration of employment [12,13]. As of the beginning of September 2021, 508,800 foreigners had valid documents entitling them to stay in Poland, of which 59.4% were men. Citizens of Ukraine prevailed in this group, accounting for more than half (55.9%) [14,15]. In the Pomeranian Voivodeship, which has a population of approx. 2.35 million people [16], until September 2021 almost 9500 residence permits were issued to Ukrainian men [15]. One can presume that the real number of migrants is higher both due to the long waiting time for a formal decision regarding the possibility of remaining legally (and, consequently, inclusion in the statistics) and to illegal immigration. Figure 1 shows the gradually increasing migration from Ukraine to Poland since the Euromaidan revolution (2014) [17] using the example of men settling in the Pomeranian Voivodeship. Similar correlations are observed in most regions of the country. Additional information is provided by the analysis of declarations of giving work to foreigners, which in January–June 2021 in the Pomeranian Voivodeship alone concerned 40,000 men from Ukraine [18,19].

Studies in other countries have found greater levels of distress in migrant and refugee populations during the COVID-19 pandemic. These populations reported subjectively higher levels of discrimination and more daily stressors [8]. However, there is a lack of research on the subjective perception of the levels of stress experienced by immigrants from Ukraine to Poland during the pandemic. Before the outbreak of COVID-19 in 2019, the high acceptance of the model of life in Poland was indicated in the study in which among immigrants from Ukraine and Belarus as many as 55.5% of the respondents declared their willingness to stay permanently in the Pomeranian Voivodeship [10]. In turn, a survey conducted in 2020 (already during the pandemic) among immigrants from Ukraine staying in Poland showed that 67% of them were satisfied with their life situation in Poland, despite the fact that 53% declared that the pandemic worsened their satisfaction with this situation. 49% of them had already brought or were planning to bring their families to Poland [20].

A major problem that may affect the level of perceived stress, especially during a pandemic that represents a substantial health hazard, is the limitation of access to medical services. Immigrants from Ukraine have a very negative opinion of access to the healthcare system in Poland; only 25% declared that it is sufficient and meets their needs. By contrast, 14% stated that access is practically impossible, 29% that it is difficult and 11% that only paid care is available to them [20]. In another study, on the problems with living conditions declared by Ukrainian immigrants, the majority of responses indicated difficulties in access to medical services (26.6%). Housing problems came second (23.2%). In spite of this, 32.4% of respondents said they had no problems. Simultaneously, a need for support in the field of medical care was declared by 26.5%, which was the 6th in order [10].

Another problem is an increase in negative attitudes towards immigrants. Regarding the Pomeranian Voivodeship in 2017, 4.1% of the surveyed immigrants from Belarus and Ukraine confirmed that they were badly treated or discriminated against, while in 2019 it was as many as 40.2% [10]. This partly corresponds to the situation in Poland, which is as follows: in 2020, 48% of Polish respondents described their attitude towards Ukrainians as positive, 43% as neutral and 9% as negative [13]. This stems from many factors. Certainly, the increase in the number of Ukrainians in Poland after 2014 is important, which means intensification of contacts, and this may translate into a greater number of negative events. Moreover, Tyma [21] draws attention to the lack of a proper response on the part of the Polish state authorities to manifestations of hatred based on nationality, etc. On the other hand, the actions of local governments in the study area are very favorable to immigrants [22,23]. Some reports also point to possible disinformation activities, one element of which is the construction of a negative image of immigration and refugees [24].

### 1.3. LGBT Community in Poland and Ukraine

Ukraine has been shown to possess a lower acceptance of the LGBT+ community than Albania and Italy [25], which in previous studies seemed to be two of the most homophobic countries [26]. It is worth noting that the decriminalization of homosexual relationships in Ukraine has taken place relatively recently, and homophobic attitudes among the population have remained high for a long time [27]. Moreover, even in 2012 Ukraine was planning to introduce a law that would punish the promotion of homosexuality with imprisonment of up to 5 years [28]. It was only in 2015 that the Verkhovna Rada of Ukraine amended the Labor Code to grant equal rights to employees without regard to their sexual orientation or gender identity [29].

More recent studies [30] place Poland and Ukraine among the least tolerant countries in Europe, which is also confirmed in other publications, both as regards Poland [31,32] and Ukraine [33,34]. From a national perspective, the situation of LGBT+ people is especially bad in small towns and rural areas. Moreover, it can be noticed that the situation of LGBT+ people in Poland varies not only in terms of the juxtaposition of large and medium cities as well as towns and villages, but also in geographical terms. For example, some Polish local governments have adopted resolutions that indirectly affect the LGBT+ community and are called “LGBT-free zones” [35], although advocates of these resolutions argue the opposite [36]. The current list of local governments that have adopted such resolutions is available in the so-called “Atlas Nienawiści” (Eng. “Atlas of Hate”) [37]. Its analysis shows that such resolutions were adopted by a large number of local governments, mainly in south-eastern Poland and partially in central Poland, and that, according to the authors of the initiative, at the beginning of 2020 these local governments covered an area of nearly 1/3 of the country, covering 1/3 of its population. In the Pomeranian Voivodeship, which is the subject of this study, no local government has adopted such a resolution, despite lobbying being undertaken.

Evidence suggests that one of the more important causes of the migration of gay, bisexual and other men who have sex with men (MSM) may be the seeking of rights and freedoms unavailable to the LGBT+ community in their home countries [38,39]. Despite the lack of legal recognition of same-sex partnerships in Poland as well as some indicators placing Poland among the most conservative European countries [30] and the reservations described above, 78% of Poles declare tolerance towards homosexual persons, with the highest percentage declared by residents of the largest cities [40]. Despite social changes taking place and the growing general tolerance and acceptance of LGBT+ persons, these people are still at a higher risk of anxiety disorders, depression and substance abuse [41], which is sometimes associated with internalized homophobia [42]. Additionally, people who happen to also be migrants and belong to the LGBT+ community seem to face more barriers in the mental health system, although there is little research on this topic [43]. They are potentially affected by two types of stigma, both that against migrants and that against sexual minorities. However, most studies show both of these phenomena separately, indicating that anti-gay structural stigma (through discriminatory laws, institutional policies and cultural norms) adversely affects the health of sexual minorities [39,44], clearly correlating it with the occurrence of depression [28]. In turn, anti-immigrant stigma negatively affects immigrants’ health [45]. However, some studies show that the phenomenon of disapproval of one’s own homosexuality decreases among migrants as they move on to a new country [46], and is largely dependent on the attitudes of the local population [47].

### 1.4. Purpose of the Study

The aim of this study was to assess the ability to deal with an external situation during the third wave of the COVID-19 pandemic in Poland [48,49] in the following groups with different stress levels: Polish heterosexual men (exposure to the pandemic and restrictions related to social isolation), heterosexual men from Ukraine (additional factors related to migration) and MSM from Ukraine. Apart from the factors present in the other two groups, MSM are also susceptible to factors related to functioning within the LGBT+ minority. Furthermore, research showed that attitudes towards MSM tend to be more negative than those towards women who have sex with women [50,51]. Estimating the level of stress in the above communities could contribute to understanding the importance of individual stressors in general well-being and to answering the question of whether the pandemic itself with all its consequences [3,52] may be a strong enough stress factor that the differences resulting from migration and/or the fact of belonging to a sexual minority would become blurred.

Finding possible differences in the level of perceived stress could constitute an intro-duction to further research aiming to identify the most important risk factors for increased levels of stress in individual groups, along with the possible association of distress with the incidence of mental disorders. This, in turn, could translate into targeted preventive and therapeutic actions, taking into account the different needs and different availability of health services for people from the studied groups. A special role could be played by some of the more prolific national and local LGBT+ advocacy and support non-governmental organizations, such as Stowarzyszenie Miłość Nie Wyklucza [Eng.: Love Does Not Exclude Association], Organizacja Pożytku Publicznego Kampania Przeciw Homofobii [Eng.: the Public Benefit Organization—Campaign Against Homophobia] or Tolerado Tricity. Despite their extensive activities, to the best of our knowledge only one NGO in Poland runs a Ukrainian section supporting migrants, which is Lambda Warsaw. On the other hand, in Poland there are also organizations supporting immigrants, such as Fundacja Nasz Wybór—Ukraiński Dom w Warszawie [Eng.: Our Choice Foundation—the Ukrainian House in Warsaw]. Its goal is to work for the benefit of Ukrainian migrants in Poland, to help them integrate into the Polish society and Polish culture and to familiarize Poles with Ukrainian culture.

The phenomenon of “minorities within minorities” can be associated with multiple exclusions (due to race, nationality, ethnicity, gender, sexual orientation, mental illness, etc.) and stigmatization even within a seemingly homogeneous group. This, in turn, can translate into increased stress and a predisposition towards the development of mental disorders. This is particularly important during the current pandemic, with all its threats and limitations. On the other hand, it is possible that the chronically increased stress to which society is subjected may reach a level so high that the differences between groups observed in pre-pandemic studies disappear. However, this may be due both to the fact that personal problems of people belonging to minorities may be obscured by the traumatizing effect of the pandemic and social isolation, and to the fact that the aforementioned social isolation through social distancing also leads to less exposure to stigmatization. Such observations could suggest a need to transfer most of the efforts and resources to eliminate the current and expected prospective effects of the current epidemiological situation. Although this study is limited to one region of Poland, it tries to draw attention to problems that go far beyond its local character.

## 2. Materials and Methods

### 2.1. Participants and Procedure

The study sample consisted of Polish and Ukrainian men of working age (18–64 years) living and working in Tri-City in Poland (an agglomeration in the Pomeranian Voivodeship comprising the cities of Gdańsk, Sopot and Gdynia, with a population of about one million people). Additionally, people living in the Tri-City suburban zone, including smaller towns located in the immediate vicinity of the agglomeration, were allowed to participate in the study. The field phase of the study (1 March–11 April 2021) was conducted at the peak of the third wave of the COVID-19 epidemic in Poland and the related restrictions on movement, social gatherings and running of a business. The exclusively male sample was selected for this study due to more stigma around homosexuality in MSM than in women who have sex with women [50,51].

The selection of respondents was deliberate; therefore, it was of a non-probabilistic selection type and was mainly based on the snowball method. In order to diversify the sample in terms of professional status and level of education, the study started by selecting different work environments dominated by people with a specific level of education. The informants who distributed the questionnaires in their environment came from professional circles related to large corporations employing highly qualified specialists, as well as from unskilled workers performing simple construction work. Some of the questionnaires were also distributed in semi-legal workers’ hostels operating in Tri-City. Some of the respondents from Ukraine were reached by establishing direct contact in a grocery store, where they did shopping before work. People who did not want to fill in the questionnaire in the presence of the researcher could do it online via a Google form, which was done by six persons.

In total, the research sample amounted to *n* = 370. Among the respondents, there were 168 people with Ukrainian citizenship and 202 with Polish citizenship. As regards the respondents’ sexual orientation, the sample included 333 heterosexual persons and 37 homosexual or bisexual persons. Combining the variables of citizenship and sexual orientation, the sample included 202 men from Poland of heterosexual orientation, 131 men of Ukrainian origin and heterosexual orientation and 37 Ukrainian men of homosexual or bisexual orientation.

### 2.2. Measures

The study was conducted using a self-reported community survey. In view of the conditions of the study, a short questionnaire consisting of 19 closed questions was used. Due to considerable distrust among the respondents and their reluctance to provide personal information, the questionnaire was shortened to 13 questions, and only these answers were included in the analysis. Questions regarding sensitive demographic data and the region (district) of origin were excluded due to a poor response rate from the first respondents during the pilot phase of the study.

The first two questions concerned citizenship (Ukrainian or Polish) and sexual orientation (heterosexual or homo-/bisexual). The third question related to the current state of health of the subject with no distinction between general and mental health (“How would you rate your current health condition?”). The possible answers were as follows: “very good”, “good”, “average”, “bad” and “very bad”. The last 10 questions were questions included in the PSS-10—Perceived Stress Scale [53] in its standard timespan version, with the scoring of items 4, 5, 7, and 8 reversed. The total score ranged from 0 to 40, with higher scores indicating higher levels of perceived stress. According to the instruction, scores ranging from 0–13 would be considered low stress, scores ranging from 14–26 would be considered moderate stress and scores ranging from 27–40 would be considered high perceived stress [54]. The following indicators were also distinguished in accordance with the suggestions of the two-factor structure by Roberti et al. [55]: F1 (perceived helplessness) and F2 (perceived self-efficacy).

In order to maintain the comparability of the results with other surveys, the calculations and analyses were also performed for the individual items of the PSS-10 scale. However, although the problem of using single-item scales and single elements of multi-item scales is known [56], the value of comparability is worth recognizing. During the COVID-19 pandemic, a similar study was conducted on the perceived level of stress among Polish and Italian residents [5].

Both the questionnaire and the PSS-10 form were in Polish. Only minor linguistic difficulties were encountered among Ukrainian citizens. In these cases, the interviewer assisted the respondent in his native language (Ukrainian, Russian) or in Surzhyk [57].

### 2.3. Ethical Consideration

Participation was voluntary and data processing was anonymous and confidential in accordance with current regulations. The research followed the rules on bioethics established in the Declaration of Helsinki and its latest update. This project was approved by the Clinical Research Ethics Committee of Medical University of Gdansk (reference number: NKBBN/144/2021).

### 2.4. Statistical Analysis

Quantitative variables were tested for compliance with the normal distribution by the Shapiro-Wilk test. Depending on the obtained result of the compliance test, appropriate statistical tests were used for intergroup comparisons. In the case of comparisons of two groups, the *t*-test for parametric analyses and the Mann-Whitney *U* test for nonparametric analyses were used, while in the case of multi-group comparisons, one-way analysis of variance (ANOVA) and the Kruskal-Wallis test were used in the case of failure to meet the parametric assumption. The Mann-Whitney *U* test was used to compare groups of unequal sizes [58]. In the case of a statistically significant result of intergroup comparisons in the ANOVA scheme, *post hoc* tests were performed in order to minimize the error resulting from unequal sample sizes (Tamhane’s T2 and Games-Howell).

To determine which groups were significantly different from one another in individual PSS items in the case of the Kruskal-Wallis test, post-hoc Dunn’s [59] tests with Holm’s [60] adjustment method were also performed.

The reliability coefficient Cronbach-*alpha* for the PSS-10 test in the entire sample was 0.926, and was above 0.92 in individual subgroups included in the analyses. The level of statistical significance in the analyses was adopted at *p* < 0.05.

At the initial stage of the conducted research, the statistical power analysis was not performed. As the power parameters were not calculated prospectively, retrospective analysis was not implemented due to the “after” approach being considered as a mistake [61,62].

Statistical analyses were performed using the R (4.0.3, Vienna, Austria) and RStudio (1.4.1103, Boston, MA, USA) software.

## 3. Results

All respondents (*n* = 370) were Caucasian men aged 18–64, living in a large urban center (the Tri-City agglomeration has over one million inhabitants) [63].

An overview of the study results, taking into account intergroup comparisons, is presented in Table 1. The level of stress measured with the PSS-10 questionnaire yielded a score of 24.91 (SD ± 9.11) for the entire sample. A low level of stress (a score of less than 13 points) was experienced by 15.4% (*n* = 57) of the respondents, a medium level of stress (a score from 14 to 26 points) by 33.0% (*n* = 122) and a high level of stress (a score over 27 points) by more than half of the study participants (51.6%, *n* = 191).

The general level of perceived stress as well as the F1 (perceived helplessness) and F2 (perceived self-efficacy) indices calculated from the PSS-10 questionnaire were not related to the respondents’ citizenship or their sexual orientation. Differences were revealed only when analyzing individual PSS-10 questions (Table 2). The level of perceived stress was also negatively correlated (rS = 0.447, *p* < 0.001) with self-assessment of the health condition. In the group of respondents assessing their health condition as very bad or bad, the mean PSS-10 test score indicated a high level of perceived stress. Respondents who rated their health as average usually obtained a PSS score revealing an experience of stress of moderate and strong intensity, and the respondents who assessed their health as good and very good obtained results indicating stress at a moderate level. No value indicating a low level of perceived stress was recorded in any of the groups distinguished on the basis of their health condition.

Reliability analysis of the PSS-10 and its two-factor structure is presented in Table 3.

## 4. Discussion

To our knowledge, this is the first study to focus on migrant LGBT+ communities during the COVID-19 pandemic, not only in the context of mental health burdens but also in general. It is also one of the few studies concentrating on perceived stress in MSM or (im)migrant populations in the course of the current COVID-19 pandemic.

Although the results obtained indicate a similar mean level of perceived stress in heterosexual citizens of Poland and Ukraine (PSS-10 scores of 24.71 and 24.77, respectively) and a higher one in MSM from Ukraine (26.49), these observations did not reach the level of statistical significance (*p* = 0.551). Similar relationships were revealed in the analysis of the two-factor model of perceived helplessness and perceived self-efficacy in all three groups. Significant differences appeared only when analyzing specific items of PSS-10. The respondents’ health condition proved to be the main factor influencing perceived stress.

Worse self-assessment of health status was associated with greater perceived stress, which is probably related to greater concerns about falling ill and the risk of a severe course of the disease during the third wave of the COVID-19 pandemic, which was ongoing during the study [49]. It may also be partially due to the differences in awareness and the previous experience of health issues and risks, but these were not explored between the groups. It is worth noting that in comparison to the period before the pandemic, there was a noticeable deterioration in the assessment of one’s own health. In 2019, 71.6% of men from the Pomeranian Voivodeship (without distinguishing by nationality) assessed their health as good or very good (vs. 57.6% in the present study), and only 8.7% as bad or very bad (vs. 17% in the present study) [64]. It should be noted especially that the data from 2019 also took into account men aged over 64 years old who assessed their health the worst. Among migrants, a negative assessment of their health condition may be an additional stress factor (not only during a pandemic) due to the significantly more difficult access to health services in their host country [65,66].

Lack of significant differences in the level of perceived stress between the three groups may be explained by the significant impact of the COVID-19 pandemic. Hypothetically, general mental health burdens caused by various factors associated with current epidemiological situation may have reached a level so high that it prevents differentiation between various risk factors. However, the informative value of this null result is questionable given that one group contained only 37 participants, meaning there was a likely quite low statistical power to have been able to detect a significant difference, even if there was one. Admittedly, there is a lack of data on the level of stress in the studied samples in the period before the pandemic or at its earlier stages. Consequently, it is difficult to assess the extent to which the values obtained reflect a gradually deteriorating functioning in or, on the contrary, an increasing adaptation to the epidemic situation. Previously conducted large studies [5] indicate, however, that the level of stress among Polish male citizens (without distinguishing between sexual orientation) was at a much lower level during the first wave of the pandemic (the average PSS-10 score at that time was 19.66 vs. 24.71 in the present study during the third wave). In addition, research on quality of life in Gdańsk (part of the studied Tri-City agglomeration) conducted in June 2021, already after the end of the third wave, on a group of 1500 people [6] indicated that nearly half of the respondents (48.1%) assessed their mental well-being as worse than before the pandemic. Another point of reference may be the recently published [64] statistical report for 2019, i.e., immediately preceding the outbreak of the pandemic, according to which depressive symptoms, as measured by the Patient Health Questionnaire (PHQ-8) [67], were manifested by 12% of men, regardless of age.

Acknowledging the limitations of probing single-elements of multi-item scales [Hoeppner], we decided to perform a more detailed analysis of the individual PSS-10 questions, looking for possible differences between the study groups. Several distinctions were observed. MSM from Ukraine, more often than respondents from other groups, indicated the feeling of being unable to control important aspects of their lives and to deal with current affairs (‘being on top of things’), as well as the feeling of being anxious or stressed. The screening nature of PSS-10 and the lack of data from before the pandemic prevent a clear conclusion as to what extent the observed dysfunctions are acute or chronic/permanent (resulting from, e.g., chronic stress caused by the intolerance experienced during the lifetime of the respondents or from constitutive personality traits) [68,69]. These men also reported a more frequent feeling of anxiety caused by sudden events and a sense of inability to control irritations in their lives, as well as a more frequent feeling of an inability to cope with the accumulated difficulties than with the group of Polish heterosexual men. Yet in these cases, even higher results, indicating greater problems concerning these aspects, were noted in heterosexual men from Ukraine. This suggests that the observed differences are more related to migration itself and to the ensuing lower stability of life and employment [70]. Especially during a pandemic, seasonal workers may be more prone to sudden job-cuts or transfers to different positions (due to shortages resulting from absenteeism connected to illnesses) [71]. In addition, people staying in night shelters, workers’ hostels etc. may also be subject to more severe pandemic restrictions in their place of residence in their free time, which is not experienced by local residents staying at that time in their family homes. Another explanation may be that sudden events causing anxiety are not directly related to the pandemic, but rather to unpleasant experiences resulting from anti-immigrant stigma also known as the ‘COVID-stigma’. Already at the beginning of 2020, the World Health Organization noted that, in view of the COVID-19 pandemic, minority groups experienced increased discrimination and stigmatization by mainstream society. Accused of posing an epidemic threat, members of national and ethnic minorities are particularly vulnerable to such negative consequences of the pandemic as stress, anxiety and depression. Research results indicate that the pandemic exacerbates discrimination and stigmatization behaviors, and reinforces economic, social and health inequalities, which leads to a deterioration in the psychological well-being of minority groups. Members of minority groups and migrants are blamed for creating epidemic threats for majority groups. Increasing discriminatory behavior, including manifestations of aggression, as well as the deteriorating situation of the labor market and the loss of sources of income, have negative psychological effects [72,73]. On the other hand, Polish heterosexual men felt less confident that they would be able to cope with their personal problems and, which may be related, they were more often angry at events beyond their control. One possible interpretation is that there is a greater sense of agency among migrants [74], who, making serious life decisions, decided to leave their homeland. It is possible that this migration, being primarily economic in nature [11], provided Ukrainian men (regardless of their sexual orientation) with the means to solve their personal (financial) problems. Another point might be that the people who possess the mental strength to migrate have a greater sense of coherence, resourcefulness and are more resilient [75,76], so that their responses are more positive. Paradoxically, increasing stigmatization may also enhance bonds within a group of migrants, especially those speaking the same language, and thus reduce the level of stress associated with acculturation [77]. For the LGBT+ group, being in a more tolerant place than their homeland could also be a relief from some personal burdens, for example by making it easier to form new relationships without a fear of exposure to harassment, as could be the case in their country of origin [28]. The above should be considered in the context of three distinct dimensions of social identification: centrality (the importance of the group to the self), ingroup affect (the value ascribed to being a member of the group) and ingroup ties (attachment to other members of the group) [77,78]. Physical and mental health outcomes are significantly associated with social identification as a positive predictor of group-derived efficacy and individual self-efficacy [79].

Moderately, respondents often reported a feeling that things were not going as planned and that they were experiencing difficulties in coping with all their responsibilities. In these aspects, no significant differences were noted between the studied groups, which may indicate that the pandemic itself, with the associated risks, nuisance and, possibly, new obligations [3], is the main reason why things do not go as the subjects would have liked.

The study comprised a high percentage of MSM from Ukraine (37 out of 168 people, i.e., 22%), compared to the expected 3–6% [80]. This may be related to the fact that, with a general reluctance to participate in the study, people whom it directly concerned, i.e., men from the LGBT+ community, were more interested in participating. It is also possible that such overrepresentation results from the fact that metropolitan areas of Poland, including the Pomeranian Voivodeship, are generally more tolerant of sexual minorities and are therefore more often migration destinations for people from LGBT+ communities [15,81].

### 4.1. Limitations

We recognize several limitations to our study, such as its cross-sectional character and a relatively small MSM sample. Despite the researchers’ efforts, the method of recruiting participants may have resulted in the fact that the study groups do not reflect the actual structure of migrants from Ukraine and of Polish citizens.

The respondents’ distrust, perhaps caused by the chosen method of recruitment and by waiting for them to complete the survey, prevented collecting important demographic data, such as education, exact age, marital status, income, current housing conditions or the place of origin. Furthermore, completing the questionnaire in the presence of the interviewer and, at times, the need for their assistance in translation may have significantly limited honesty of the responses in some cases. In the context of the third wave of the pandemic, housing conditions may have played a special role. Ukrainian seasonal and lower-skilled workers often stayed in workers’ hostels or dormitories, which exposed them to more contact and was associated with a greater risk of contracting COVID-19. This infection, in view of the difficulty of access to medical services [66], could be associated with greater health complications for migrants than for Polish citizens. Therefore, this group would be more exposed to stress. There is also a lack of information on medical history, which, in the case of some chronic diseases, could be associated with a worse course of COVID-19, and therefore generate greater anxiety in at-risk participants of the study. The participants’ knowledge of specific health risks and their previous experience in the different groups were not determined, nor were inclusion and exclusion criteria based on medical history used in the study, so that there remains a potential for confounding factors associated with such knowledge.

There are also no data on the length of migrants’ stay in Poland (and the migrants’ generation), the reason for migration, the level of bonds with the country of origin and religion, i.e., factors that could have a significant impact on the level of perceived stress (integration, acculturation, language skills) [46]. However, it should be remembered that many Ukrainian migrants probably work in Poland illegally, and that they came from a country where, formally, a war is still being waged [82]. Moreover, MSM from Ukraine could experience various kinds of violence in their country of origin. Considering the above, it seems that the obtained sample is still satisfactory considering such a short period of study (the third wave of the COVID-19 pandemic), and the number of MSM coming from Ukraine is quite substantial.

Another limitation is the lack of a control group for Polish MSM, which would probably allow distinguishing factors more related to sexual orientation than those correlated with migration. Moreover, the entire survey was conducted only in Polish, partly due to the fact that in Ukraine two languages and Surzhyk are used in close relation to the region of origin, and their use is often a source of conflict [83]. With the expected high level of distrust, we wanted to avoid presenting the participant with the form in a language that they did not acknowledge and which might have raised their additional reluctance.

Finally, one should take into account the lack of previous similar studies that could serve as a reference point for the interpretation of the obtained data.

### 4.2. Future Outlook

The next study prepared by the present authors, which is to be conducted during the fourth wave of the pandemic, will attempt to address most of the above-mentioned limitations. Due to the lack of any similar research, it would seem reasonable to assess the level of stress among LGBT+ migrants in other parts of the world, especially with additional consideration of ethnic minorities, which seem to be even more exposed to stress [84] than the ethnically homogeneous group of Ukrainian migrants described in this study.

## 5. Conclusions

In this study, we assessed the level of perceived stress among men during the third wave of the COVID-19 pandemic. The groups of Polish citizens living in the Tricity agglomeration as well as migrants from Ukraine, were compared, with the latter being broken down into heterosexual and homosexual and bisexual men, the hypothesis being that migrants, especially migrants belonging to a sexual minority, will be exposed to higher stress due to numerous factors that may adversely affect their daily functioning. The analysis of the survey among 370 respondents did not show statistically significant differences between the three studied groups in their general level of perceived stress measured by PSS-10, but it did reveal numerous differences in coping with various aspects of everyday functioning between these groups. Negative assessment of one’s own health proved to be the main factor negatively affecting the level of perceived stress. To our knowledge, this is the first study to assess migrants belonging to a sexual minority during the COVID-19 pandemic.

Further research should focus on recruiting a larger group of respondents and observing them during the subsequent stages of the pandemic, both in the periods of its exacerbation and decline (and the accompanying reduction in the number of restrictions), as well as on identifying factors negatively affecting the level of stress among the groups of respondents. In this research, it would also be advisable to consider other regions of the world as well as ethnic minorities among migrants and LGBT+ communities. Regardless of this, our research shows differences in the needs, resources and ways of coping with stress between men who are Polish citizens and migrants from Ukraine, both heterosexual and belonging to the MSM group.

These varying needs could be addressed by psychological and social interventions from various NGOs. Such interventions, apart from direct support, may focus on limiting the gap in the accessibility of health services (including mental health care services). Furthermore, when considering migrant workers, one should acknowledge varying insurance statuses. Legal workers pay contributions to the National Health Fund, and theoretically have the same access to health benefits as Polish citizens. Some employees rely only on private insurance, and some are not insured at all because they work outside the system. In the first two cases, it would be in the interest of the payer/insurance company to take preventive measures to reduce high levels of stress and thus reduce the risk of developing more serious psychiatric disorders that would require specialized, cost-intensive clinics or hospital psychiatric treatment. Uninsured persons cannot count on free help and are left to use private health care, which may result in their delaying looking for help in order to avoid incurring costs. Such a phenomenon of neglecting one’s mental health may result in serious consequences, including suicide due to untreated depressive disorders.

Undoubtedly, a comprehensive approach to the problem of mental disorders among various minorities is necessary, both in research work and in preventive and therapeutic activities. In addition to the human factor, more thorough studies should take into ac-count cost-benefit economic analyses that could convince the authorities to devote more attention to this issue.

## Figures and Tables

**Table 1:** PSS test results crossed by independent variables.

	*n* (%)	PSS Score Mean ± SD	*p*-Value
**total**	370 (100%)	24.91 ± 9.11	-
**citizenship**			
Polish	202 (54.6%)	24.71 ± 9.01	0.545
Ukrainian	168 (45.4%)	25.15 ± 9.23	
**orientation**			
heterosexual	333 (90.0%)	24.74 ± 9.16	0.286
homo- or bisexual	37 (10.0%)	26.49 ± 8.62	
**citizenship and orientation**			
heterosexual citizens of Poland	202 (54.6%)	24.71 ± 9.01	0.551
heterosexual citizens of Ukraine	131 (35.4%)	24.77 ± 9.41	
homosexual or bisexual citizens of Ukraine	37 (10.0%)	26.49 ± 8.62	
**health condition self-assessed by the respondents**			
very bad and bad	63 (17.0%)	30.54 ± 7.56	<0.001
average	94 (25.4%)	26.98 ± 8.40	
good and very good	213 (57.6%)	22.33 ± 8.88	

**Table 2:** Descriptive statistics of PSS-10, F1 and F2.

PSS-10 Item	Heterosexual Citizens of Poland	Heterosexual Citizens of Ukraine	Homosexual or Bisexual Citizens of Ukraine	Kruskal–Wallis	*p*-Value
	mean ± SD			H	
1. Been upset	2.65 ± 1.15	3.11 ± 0.97	3.05 ± 0.91	15.284	<0.001
2. Unable to control	2.6 ± 1.07	2.67 ± 1.14	3.27 ± 0.87	14.710	<0.005
3. Nervous-stressed	2.6 ± 1.05	2.25 ± 1.29	3.27 ± 0.77	23.157	<0.001
4. Felt confident (R)	2.63 ± 1.16	1.6 ± 1.29	1.68 ± 1.36	60.317	<0.001
5. Things-your way (R)	2.54 ± 1.14	2.35 ± 1.34	2.59 ± 1.3	1.692	0.429
6. Could not cope	2.63 ± 1.11	2.53 ± 1.24	2.43 ± 1.07	1.384	0.501
7. Control irritations (R)	1.98 ± 1.13	2.67 ± 1.08	2.46 ± 1.43	29.430	<0.001
8. On top of things (R)	1.93 ± 1.15	2.59 ± 1.15	2.89 ± 0.99	40.697	<0.001
9. Been angered	2.83 ± 0.96	2.25 ± 1.17	2.3 ± 1.39	18.175	<0.001
10. Could not overcome	2.3 ± 1.16	2.73 ± 1.26	2.54 ± 0.99	14.579	<0.005
PSS-10 total score	24.71 ± 9.01	24.77 ± 9.41	26.49 ± 8.62	1.190	0.551
F1	15.63 ± 5.55	15.56 ± 5.77	16.86 ± 9.62	1.291	0.524
F2	9.08 ± 3.88	9.21 ± 3.97	9.62 ± 4.35	0.636	0.728

R = item is reverse scored; F1 = perceived helplessness; F2 = perceived self-efficacy; SD = standard deviation; PSS-10 = 10-item perceived stress scale.

**Table 3:** Reliability analysis of PSS-10, F1 and F2.

	Heterosexual Citizens of Poland	Heterosexual Citizens of Ukraine	Homosexual or Bisexual Citizens of Ukraine
F1	0.925	0.898	0.874
F2	0.868	0.833	0.871
PSS-10	0.942	0.932	0.920

## Data Availability

The raw data supporting the conclusions of this article will be made available by the authors, without undue reservation.
